# Duration of face down positioning following full-thickness macular hole repair: A protocol for a randomized pilot study

**DOI:** 10.1371/journal.pone.0304566

**Published:** 2024-08-20

**Authors:** Keean Nanji, Paola Lourdes Oquendo, Sangeetha Srinivasan, Chinmayi Vyas, Femin Prasad, Forough Farrokhyar, Varun Chaudhary

**Affiliations:** 1 Division of Ophthalmology, Department of Surgery, McMaster University, Hamilton, Ontario, Canada; 2 Department of Health Research Methods, Evidence and Impact, McMaster University, Hamilton, Ontario, Canada; Akita University: Akita Daigaku, JAPAN

## Abstract

**Objective:**

Full-thickness macular holes (FTMH) are defects in the fovea involving all neural retinal layers. They reduce patients’ visual acuity (VA) and impact their quality of life. FTMHs are repaired with pars plana vitrectomy (PPV) with intraocular gas tamponade and post-operative face-down positioning (FDP). There is no consensus regarding the ideal positioning requirements following FTMH repair and there lacks clear guidelines on the topic. While analysis of global practice patterns indicates that between 5–7 days is the most common duration suggested by surgeons, there is significant heterogeneity in surgeon preferences. There is, however, biological plausibility to support minimal or even no FDP following surgery and given the disabling nature of FDP for patients, there is a need to better assess key patient outcomes with different FDP durations. As such, this prospective randomized controlled pilot trial will compare 3-days of FDP to 7-days of FDP following PPV for FTMH.

**Methods:**

This single-centered, parallel-group randomized controlled pilot trial will randomize patients 1:1 following PPV to 3 days or 7 days of FDP. This investigation has been approved by the local ethics board (HiREB # 16100) and has been registered on clinicaltrials.gov (NCT06000111). The primary objective will be focused on assessing the feasibility of a larger trial; this will be determined through an assessment of the recruitment rate, retention rate, completion rate and recruitment time. The secondary outcomes involve assessment of the following patient-important outcomes a) macular hole closure rate, b) best-recorded VA, c) a general quality of life measure and vision-specific quality of life measure, d) patient compliance and e) complication rates. Outcomes will be evaluated at 3 months following surgery.

**Discussion:**

The results of this pilot study will determine the feasibility of a larger-scale trial that will answer a patient important question with clinical equipoise.

## Introduction

### Background and rationale

Full-thickness macular holes (FTMH) are defects in the fovea involving all neural retinal layers [1]. They reduce patients’ visual acuity (VA) and have a significant impact on patients’ quality of life [2]. Left untreated, patients experience progressive loss of central visual acuity [3]. The current standard of repair for FTMHs is pars plana vitrectomy (PPV) with or without peeling of the internal limiting membrane (ILM), the use of intraocular gas tamponade and post-operative face down positioning (FDP) [1, 3].

Face-down positioning (FDP) is the standard recovery posture for patients post PPV for FTMH. This however, can be physically challenging considering idiopathic FTMH occur most frequently in the elderly [4]. Previous investigations have demonstrated that over 50 percent of patients have described FDP as either difficult or very difficult [5, 6]. Moreover, rare but serious systemic complications such as pulmonary embolism or ulnar nerve palsies have been reported after FDP [7]. Consequently, suggestions have been made to reduce the duration of FDP or eliminate FDP completely [8, 9]. Presently, there is no consensus regarding the ideal positioning requirements following FTMH repair. A 2020 Preference and Trends Survey performed by the American Society of Retina Specialists (ASRS) and completed by 1273 retinal specialists from around the world demonstrated significant variability in current practices [10]. While the vast majority of surgeons recommend FDP, the duration of FDP varied considerably; the most frequently recommended duration was between 5–7 days of FDP (39.5% of responses) followed by 3–4 days (29.3% of responses).

There is, however, biological plausibility to support minimal or no FDP following surgery. The rationale for FDP is to ensure that the FTMH can be isolated from intraocular fluid thereby permitting subfoveal fluid absorption and closure of the FTMH [2, 11]. However, previous investigations have demonstrated that the first 24 hours is the most important period for isolating the FTMH from the intraocular fluid [2]. A large gas bubble even without FDP would be sufficient to achieve this [2]. In keeping with this, a recent meta-analysis of randomized controlled trials demonstrated no difference between FDP and non-FDP in FTMH closure [12]. In this investigation, there was a visual benefit to FDP however the confidence intervals included values of trivial clinical significance and there were serious concerns of imprecision. Consequently, the authors concluded that there is a need for a future large, well-conducted trial evaluating patient-important outcomes following FTMH repair [12].

Previous investigations evaluating the quality of life outcomes have demonstrated that patients prefer non-FDP; Veith et al. found that patients rated FDP as significantly more uncomfortable compared to non-FDP (p = 0.010) and that patients found FDP to significantly impair sleep quality (p = 0.001) [13]. Tadayoni et al. found that when they asked patients to rate the painfulness of positioning on a visual analog scale (0–10), pain was rated 6.52 ± 2.48 and 2.53 ± 2.6 in the FDP and alleviated positioning group respectively [7]. Patient preferences are also reflected by the fact that positioning compliance has been demonstrated to be lower in FDP groups compared to non-FDP [7], and by the fact that positioning compliance following PPV declines over time [14]. An investigation examining compliance during the first 3 days post-operatively found significantly decreased compliance each day after surgery [14].

Given the lack of guidelines on the topic, the corresponding heterogeneity in practice patterns, the lack of biologic rationale, and the disabling nature of FDP for patients, there is a need to better assess key patient outcomes with different FDP durations. As such, this investigation through a prospective randomized controlled pilot trial will compare 3-days of FDP to 7-days of FDP following PPV for FTMH. A pilot trial was deemed necessary to best assess the feasibility of a larger trial and to understand the resource and management requirements of the future larger-scale trial [15]. Pilot randomized trials are a type of feasibility study and are smaller-scale versions of the main study [16]. Pilot trials have been shown to increase the likelihood of a successful man trial and improve the methodology of the main study [15, 16].

The population examined in this investigation will be consecutive patients who present with an idiopathic FTMH to the Hamilton Regional Eye Institute. The intervention will be PPV with ILM peel followed by 3-days of FDP which will be compared to the control group undergoing PPV with ILM peel followed by 7-days of FDP. The primary outcome will be the feasibility of completing a larger-scale trial. Secondary outcomes will be focused on functional, structural, and quality-of-life outcomes. All outcomes will be examined at 3 months post-surgery.

### Objectives

Our primary objective will be focused on assessing feasibility. Feasibility will be assessed by measuring the recruitment rate, retention rate, and completion rate at 3 months following surgical repair as well as the recruitment time.

Our secondary outcomes will include a) macular hole closure rate at 3 months following surgery; two independent readers, masked to treatment allocation, will independently grade the outcome in each instance as closed, or open. Outcomes classified as open will be further divided into open and flat (without a cuff of subretinal fluid), or open and elevated (with a cuff of subretinal fluid). b) best-recorded visual acuity at 3 months post-surgery, c) compliance with FDP as assessed utilizing self-administered questionnaires four times per day during the duration of FDP, d) patient self-administered vision-specific validated quality of life measure, the 25-item National Eye Institute Visual Function Questionnaire (NEI VFQ-25) and a validated quality of life measure, the Quality of Life Scale (QOLS), and e) complication rates (endophthalmitis, retinal detachment, vitreous hemorrhage) as assessed by a masked physician.

The interpretation of findings will be: a) the full-scale trial is feasible with no changes to the protocol; b) the full-scale trial is feasible with small changes to the protocol; c) the full-scale trial is infeasible. The study will be considered feasible if the retention and completion rates are > 80% of randomized patients. The study will be considered feasible with small changes if either the retention or completion rate is <80% of randomized patients and the study will be considered infeasible if the completion and retention rates are <80% of randomized patients.

### Trial design

This study will be a single-centered, parallel group randomized controlled pilot trial. Patients will be randomized 1:1 between groups.

## Methods

### Study setting

The study will be conducted at the Hamilton Regional Eye Institute located at St. Joseph’s Hospital in Stoney Creek Ontario. The study protocol is reported in line with the Standard Protocol Items: Recommendations for Interventional Trials (SPIRIT) statement (Table 1) [17]. Ethics approval for this project was granted by the Hamilton Integrated Research Ethics Board (HiREB project ID: 16100). This trial has been registered on clinicaltrials.gov (NCT NCT06000111). This study will adhere to the tenets of the Declaration of Helsinki.

**Table 1:** SPIRIT checklist.

Section/item	Item No	Description	Page Number
**Administrative information**			
Title	1	Descriptive title identifying the study design, population, interventions, and, if applicable, trial acronym	1
Trial registration	2a	Trial identifier and registry name. If not yet registered, name of intended registry	1
	2b	All items from the World Health Organization Trial Registration Data Set	1
Protocol version	3	Date and version identifier	1
Funding	4	Sources and types of financial, material, and other support	1
Roles and responsibilities	5a	Names, affiliations, and roles of protocol contributors	1, 17
	5b	Name and contact information for the trial sponsor	N/A
	5c	Role of study sponsor and funders, if any, in study design; collection, management, analysis, and interpretation of data; writing of the report; and the decision to submit the report for publication, including whether they will have ultimate authority over any of these activities	N/A
	5d	Composition, roles, and responsibilities of the coordinating centre, steering committee, endpoint adjudication committee, data management team, and other individuals or groups overseeing the trial, if applicable (see Item 21a for data monitoring committee)	N/A
**Introduction**			4
Background and rationale	6a	Description of research question and justification for undertaking the trial, including summary of relevant studies (published and unpublished) examining benefits and harms for each intervention	4
	6b	Explanation for choice of comparators	4,5,9
Objectives	7	Specific objectives or hypotheses	6,7
Trial design	8	Description of trial design including type of trial (eg, parallel group, crossover, factorial, single group), allocation ratio, and framework (eg, superiority, equivalence, noninferiority, exploratory)	7
**Methods: Participants, interventions, and outcomes**			
Study setting	9	Description of study settings (eg, community clinic, academic hospital) and list of countries where data will be collected. Reference to where list of study sites can be obtained	8
Eligibility criteria	10	Inclusion and exclusion criteria for participants. If applicable, eligibility criteria for study centres and individuals who will perform the interventions (eg, surgeons, psychotherapists)	8
Interventions	11a	Interventions for each group with sufficient detail to allow replication, including how and when they will be administered	8,13
	11b	Criteria for discontinuing or modifying allocated interventions for a given trial participant (eg, drug dose change in response to harms, participant request, or improving/worsening disease)	13,14
	11c	Strategies to improve adherence to intervention protocols, and any procedures for monitoring adherence (eg, drug tablet return, laboratory tests)	13,14
	11d	Relevant concomitant care and interventions that are permitted or prohibited during the trial	N/A
Outcomes	12	Primary, secondary, and other outcomes, including the specific measurement variable (eg, systolic blood pressure), analysis metric (eg, change from baseline, final value, time to event), method of aggregation (eg, median, proportion), and time point for each outcome. Explanation of the clinical relevance of chosen efficacy and harm outcomes is strongly recommended	6,7
Participant timeline	13	Time schedule of enrolment, interventions (including any run-ins and washouts), assessments, and visits for participants. A schematic diagram is highly recommended (see Figure)	16,17
Sample size	14	Estimated number of participants needed to achieve study objectives and how it was determined, including clinical and statistical assumptions supporting any sample size calculations	16
Recruitment	15	Strategies for achieving adequate participant enrolment to reach target sample size	17
**Methods: Assignment of interventions (for controlled trials)**			
Allocation:			
Sequence generation	16a	Method of generating the allocation sequence (eg, computer-generated random numbers), and list of any factors for stratification. To reduce predictability of a random sequence, details of any planned restriction (eg, blocking) should be provided in a separate document that is unavailable to those who enrol participants or assign interventions	17,18
Allocation concealment mechanism	16b	Mechanism of implementing the allocation sequence (eg, central telephone; sequentially numbered, opaque, sealed envelopes), describing any steps to conceal the sequence until interventions are assigned	17, 18
Implementation	16c	Who will generate the allocation sequence, who will enrol participants, and who will assign participants to interventions	17,18
Blinding (masking)	17a	Who will be blinded after assignment to interventions (eg, trial participants, care providers, outcome assessors, data analysts), and how	17, 18
	17b	If blinded, circumstances under which unblinding is permissible, and procedure for revealing a participant’s allocated intervention during the trial	N/A
**Methods: Data collection, management, and analysis**			
Data collection methods	18a	Plans for assessment and collection of outcome, baseline, and other trial data, including any related processes to promote data quality (eg, duplicate measurements, training of assessors) and a description of study instruments (eg, questionnaires, laboratory tests) along with their reliability and validity, if known. Reference to where data collection forms can be found, if not in the protocol	15
	18b	Plans to promote participant retention and complete follow-up, including list of any outcome data to be collected for participants who discontinue or deviate from intervention protocols	15
Data management	19	Plans for data entry, coding, security, and storage, including any related processes to promote data quality (eg, double data entry; range checks for data values). Reference to where details of data management procedures can be found, if not in the protocol	15
Statistical methods	20a	Statistical methods for analysing primary and secondary outcomes. Reference to where other details of the statistical analysis plan can be found, if not in the protocol	18, 19
	20b	Methods for any additional analyses (eg, subgroup and adjusted analyses)	N/A
	20c	Definition of analysis population relating to protocol non-adherence (eg, as randomised analysis), and any statistical methods to handle missing data (eg, multiple imputation)	18, 19
**Methods: Monitoring**			
Data monitoring	21a	Composition of data monitoring committee (DMC); summary of its role and reporting structure; statement of whether it is independent from the sponsor and competing interests; and reference to where further details about its charter can be found, if not in the protocol. Alternatively, an explanation of why a DMC is not needed	19
	21b	Description of any interim analyses and stopping guidelines, including who will have access to these interim results and make the final decision to terminate the trial	19
Harms	22	Plans for collecting, assessing, reporting, and managing solicited and spontaneously reported adverse events and other unintended effects of trial interventions or trial conduct	7,19
Auditing	23	Frequency and procedures for auditing trial conduct, if any, and whether the process will be independent from investigators and the sponsor	N/A
**Ethics and dissemination**			
Research ethics approval	24	Plans for seeking research ethics committee/institutional review board (REC/IRB) approval	8
Protocol amendments	25	Plans for communicating important protocol modifications (eg, changes to eligibility criteria, outcomes, analyses) to relevant parties (eg, investigators, REC/IRBs, trial participants, trial registries, journals, regulators)	N/A
Consent or assent	26a	Who will obtain informed consent or assent from potential trial participants or authorised surrogates, and how (see Item 32)	16,17
	26b	Additional consent provisions for collection and use of participant data and biological specimens in ancillary studies, if applicable	N/A
Confidentiality	27	How personal information about potential and enrolled participants will be collected, shared, and maintained in order to protect confidentiality before, during, and after the trial	15,16
Declaration of interests	28	Financial and other competing interests for principal investigators for the overall trial and each study site	21,22
Access to data	29	Statement of who will have access to the final trial dataset, and disclosure of contractual agreements that limit such access for investigators	22
Ancillary and post-trial care	30	Provisions, if any, for ancillary and post-trial care, and for compensation to those who suffer harm from trial participation	N/A
Dissemination policy	31a	Plans for investigators and sponsor to communicate trial results to participants, healthcare professionals, the public, and other relevant groups (eg, via publication, reporting in results databases, or other data sharing arrangements), including any publication restrictions	22
	31b	Authorship eligibility guidelines and any intended use of professional writers	22
	31c	Plans, if any, for granting public access to the full protocol, participant-level dataset, and statistical code	N/A
**Appendices**			
Informed consent materials	32	Model consent form and other related documentation given to participants and authorised surrogates	N/A
Biological specimens	33	Plans for collection, laboratory evaluation, and storage of biological specimens for genetic or molecular analysis in the current trial and for future use in ancillary studies, if applicable	N/A

### Eligibility criteria

All consecutive treatment naïve patients with an idiopathic FTMH with a symptom duration of less than 6 months who agree to participate will be included in this investigation. Patients with macular hole minimum diameter >1000 μm, a history of high myopia (> -6), traumatic macular hole, amblyopia, retinal vein occlusion, inflammatory eye diseases, or who were found to have a retinal tear during either the pre-operative assessment or intraoperatively will be excluded.

### Surgical technique

In both groups, patients will undergo standard three-port, 25-gauge PPV by one experienced retina specialist (Dr. VC). If epiretinal membranes are present, they will be stained and peeled. The internal limiting membrane (ILM) will be stained with brilliant blue G dye and peeled as part of the surgical procedure. The size of the ILM peel will be approximately 1 disc diameter in radius and will increase proportionally to the size of the hole with larger peels performed for larger holes. Fluid-air exchange and a complete air-gas exchange will be performed utilizing sulfur hexafluoride (SF_6_ 18%) gas. The patients in both groups will be advised to posture face down immediately following surgery.

### Interventions

Patients will be randomized 1:1 to the 3-day FDP and 7-day FDP groups. Patients will be advised to maintain the FDP for 50 minutes of each hour. They will be advised that during their 10-minute break each hour, they should avoid face-up positioning. FDP will be advised during both waking and sleeping hours. Seven days of FDP was chosen for the control group as this is the current duration advised to patients at our institution following FTMH repair. Moreover, as per the 2020 survey conducted by the ASRS evaluating the recommended duration of FDP following FTMH, this duration falls within the 5–7 day range that is most commonly utilized by retinal surgeons around the world [10]. Three days of FDP was chosen for the intervention group as in the aforementioned recent systematic review and meta-analysis on the topic, 3 days of positioning was the most frequently utilized duration when evaluating studies advising posturing positions less than 7 days [12]. Additionally, 3 days of positioning falls within the 3–4 day range which was the second most frequently recorded response in the recent ASRS survey [10]. As such comparing these two durations has the most clinical equipoise and has the potential to be the most clinically impactful.

There are no pre-specified criteria for discontinuing or modifying allocated interventions for the included trial participants. Patients will be informed regarding their allocation immediately following surgery. Patients will have an appointment with the treating team the day following surgery at which point all post-operative care including duration of positioning will be reinforced to help improve adherence. Patient adherence to their assigned positioning regimen will be self-assessed four times per day.

### Data collection methods

After enrollment, patients will undergo standardized comprehensive ophthalmic examination including slit lamp examination, VA assessment, and measurement of intraocular pressure. VA will be measured with Snellen visual acuity with the patient’s corrective lenses with pinholing to minimize the effects of refractive error. Standardized examinations along with OCT (either the Cirrus HD-OCT, Carl Zeiss Meditec, Dublin Ca or the Heidelberg Spectralis OCT, Franklin, MA depending on the availability to the research assistant) will be performed within the 2-weeks before surgery and at the following post-operative times: 1-month, and 3-months. Patients will complete the NEI-VFQ-25 and the QOLS measures at baseline, and at 3-months following surgery. Compliance will be assessed utilizing a self-administered questionnaire in which patients will be asked to rate their compliance on a scale of 1–10 upon waking (to assess the overnight period), in the morning, at midday and in the evening. Complication rates, including rates of retinal detachment, endophthalmitis and vitreous hemorrhage will be assessed by a physician at the 1-week, 1-month, and 3-month follow-up assessments.

### Data management

Data will be collected utilizing the secure online platform, Research Electronic Data Capture (REDCap) [18]. Patients will be assigned a unique study number for which their information will be linked to. In addition to the information that will be collected as part of each ophthalmic examination, the following will be collected: the patient’s age, sex, duration of symptoms, past medical history, and past ocular history.

### Sample size

The primary outcome of this investigation is to assess feasibility, as such, a power calculation to determine the sample size was not performed [15, 19, 20]. A literature review was undertaken to help inform the optimal sample size; no consensus was found with recommendations ranging from 24 to 50 described in the literature [21–23]. A previous RCT evaluating different positioning following PPV for FTMHs experienced a 10.2% attrition and thus we similarly estimate a 10% attrition rate in this investigation [24]. As such accounting for this attrition rate, utilizing a sample size of 40 patients, we will be able to estimate an 80% retention and completion rate to within a 95% confidence interval of +/-12.4% and a 33% recruitment rate to within a 95% confidence interval of +/- 14.6% [23]. We estimate based on previous rates of FTMH at our institution that there will be 3 new eligible patients each week. Previous studies at our institution have experienced a recruitment rate of 25%. Thus, we estimate that it will take approximately 53 weeks to achieve our target sample size.

### Participant timeline

Patients will be screened for eligibility into the trial at the time of FTMH diagnosis. Eligible patients will be identified by the trial’s supervising investigator (Dr. VC) and will be introduced to the study at this time point. If the patient expresses interest in partaking in the study, they will be referred to one of the trial’s research assistants. This research assistant will not be involved in any aspect of the patient’s clinical care. The research assistant will review all details of the investigation with the eligible patient and will carry out the informed consent process including re-review all potential risks and benefits of enrolling in the trial with the patient. Interested eligible patients will subsequently sign an informed consent form before enrollment. During the enrollment visit, patients will undergo a complete ophthalmic examination and will have a macula OCT image taken. Additionally, patients will be provided with a vision-specific quality-of-life measure (NEI-VFQ 25) and a general quality-of-life measure (QOLS) to be completed before surgery.

**Fig 1:**
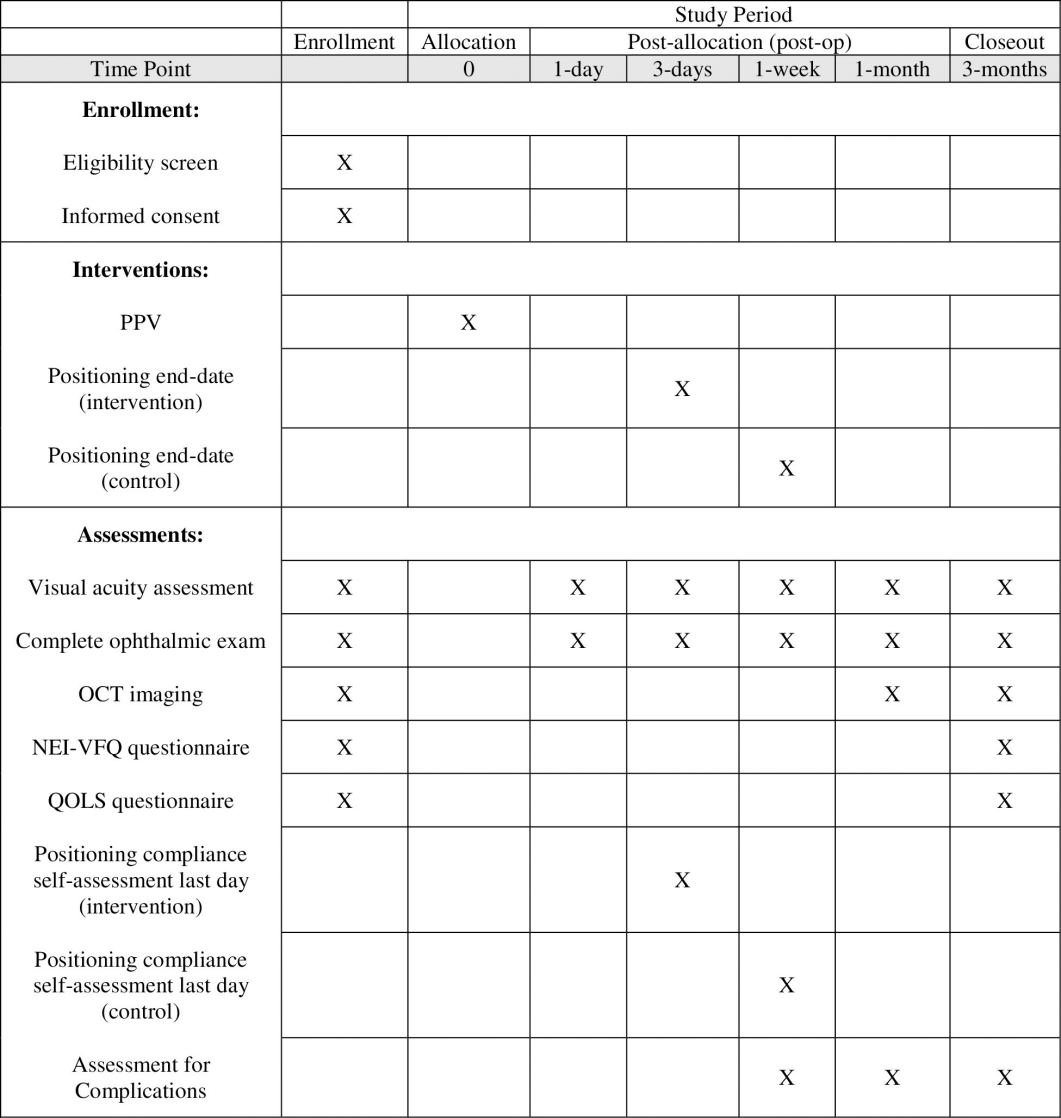
SPIRIT figure summarizing the participant timeline. Abbreviations: PPV: pars-plana vitrectomy; OCT: optical coherence tomography; NEI-VFQ: National Eye Institute Visual Function Questionnaire’ QOLS: Quality of Life Scale.

Patients will undergo PPV as outlined in the *surgical technique* section and following surgery will be randomized to either the intervention (3 days of FDP) or the control (7 days of FDP) group. Patients will be informed of their allocation following surgery while in the post-anesthesia care unit. As per our institution’s current practice patterns, patients will have scheduled follow-up appointments 1 day, 1 week, 1 month and 3 months following surgery. Additionally, as per current practice patterns, patients may have additional follow-up appointments at the attending physician’s discretion. Fig 1 summarizes the participant timeline.

### Recruitment

Participant recruitment will take place at the Hamilton Regional Eye Institute at the time of FTMH diagnosis. All patients will undergo OCT evaluation to ensure that they meet the study eligibility criteria. Eligible patients be informed regarding the risks, benefits, and alternatives to the surgical procedure and the different post-operative positioning durations. All patients will sign an informed consent form before enrollment into the study and before any measurement related to the study will be performed.

### Randomization and masking

Patients will be randomized 1:1 to the 3-day FDP or 7-day FDP groups utilizing permuted block randomization with blocks of 4, 6, and 8. Sequence generation will be performed in R (version 4.3.1, Vienna, Austria) by a research team member with experience generating lists for RCTs. The list will subsequently be uploaded to the online secure research platform Research Electronic Data Capture (REDCap) and allocation concealment will be maintained utilizing REDCap. Randomization will be performed immediately following completion of the surgical case and the surgeon will be immediately made aware of the patient’s allocation. At this point, the surgeon will inform the patient in the recovery room regarding their treatment allocation. Allocation concealment will be maintained utilizing REDCap. The randomization list will be made available to the remaining study team following the completion of the study.

All staff performing VA assessments and obtaining OCT images will be masked to treatment allocation. OCT grading of FTMH closure will be performed by two masked readers and disagreements will be resolved by a third masked reader. The participants themselves and the clinical teams managing their care will be unmasked.

### Study timeline and status

Ethics approval for this investigation was granted in September 2023. The research assistants for the project were subsequently trained and recruitment began on December 1^st^, 2023. Recruitment is ongoing and is expected to be complete by December 31^st^, 2024. Data collection is estimated to continue until March 2025. Data analysis and manuscript preparation is estimated to occur in the summer of 2025 at which point the manuscript will be submitted to a peer-reviewed journal. Following study completion, the results of this pilot trial will be reviewed, and a decision will be made regarding whether to proceed with a future single or multi-centered trial.

### Statistical methods and data analysis

Regarding the primary outcomes, the recruitment rate will be calculated as the number of patients randomized / the number of eligible patients screened. The retention rate will be defined as the number of patients who complete the follow-up measures at 3 months / the number of enrolled participants. Completion rates for data collection will be defined as the number of complete datasets for each of the outcome measures at 3 months / number of participants enrolled in the study. The recruitment time will be defined as the time taken in days to recruit 40 participants from the start of the enrollment period.

For the secondary outcomes, analyses will be performed on an intention-to-treat basis. Continuous variables will be expressed as means (± standard deviation) and categorical variables will be expressed as percentages. Continuous variables will be compared utilizing the student’s t-test or Mann-Whitney U test based on normality. Categorial variables will be compared utilizing the Chi-Squared Test. Fisher’s Exact Tests were performed for expected values less than five. A p-value of <0.05 will be considered statistically significant. All estimations will be reported with 95% confidence intervals. All analyses will be performed using R (version 4.3.1; R Foundation, Vienna, Austria).

### Data monitoring

In keeping with previous similar investigations [24], no formal Data Monitoring Committee has been created given the relatively short time span of follow-up, and minimal clinical risks. However, an interim assessment will be performed once recruitment is 50% complete. If at this point the difference in FTMH closure rates between groups crosses the 95% confidence interval of the clinically significant noninferiority margin of 15% as determined by previous investigations assessing FTMH closure [7, 24], the study will be terminated.

## Discussion

There is a gap in the current literature evaluating the optimal duration of FDP following FTMH repair. This is evident by the heterogeneity in practice patterns, lack of clear guidelines, lack of biologic rationale, and imprecision in pooled estimates in meta-analysis on the topic [12]. Moreover, FDP can be disabling for patients; [12] patient values and preferences must be taken into consideration with the current evidence when making decisions in clinical practice [25]. There is a need for a large well-conducted randomized controlled trial evaluating key outcomes to surgeons and patients including patient-reported outcome measures.

Pilot trials are an underutilized method of assessing the feasibility of large, expensive full-scale studies and can greatly increase the likelihood of success as well as minimize potential sources of waste in the main trial [15, 16]. Moreover, a pilot trial will provide great insight into the resource and management requirements of the future larger-scale trial [15, 16].

The strengths of this protocol include the multiple measures introduced to limit potential sources of bias. Firstly, randomization will occur in permuted blocks of 4,6, and 8 to protect against selection bias. Individuals involved in identifying patients and obtaining consent will not be involved in the sequence generation of the randomization list. Additionally, a single highly experienced surgeon (Dr. VC) will be performing PPV in both groups and will be masked to the patient’s treatment allocation at the time of surgery. While an element of bias is inevitable given that it will not be possible to blind patients to their allocation, we do not anticipate that this will affect our primary objectives of determining the feasibility of a larger-scale trial. Moreover, for the future main trial, the FTMH closure rate will be graded by two masked assessors and visual acuity will be assessed by a member of the research team masked to the patient’s treatment allocation. International experts in retinal diseases ranked FTMH closure rate and postoperative visual acuity as the two most important outcomes pertaining to FTMH repair [12]. Consequently, the proposed methodology ensures that all potential sources of bias pertaining to the assessment of these two outcomes are minimized. Lastly, analyses will be performed on an intention-to-treat basis to best preserve randomization and prognostic balance in study arms and minimize the type I error rate.

The limitations of the proposed investigation primarily pertain to the inherent limitations of pilot trials; namely, that the patient important research question is not answered with this investigation. However, pilot trials have repeatedly been demonstrated to facilitate a high-quality subsequent trial and we believe that this approach optimizes the chance of a successful rigorous definitive trial [15, 16, 26, 27].

In conclusion, this protocol outlines the detailed methodology for a pilot RCT that will determine the feasibility of a future larger-scale trial comparing 3 days to 7 days of FDP following FTMH repair. The main trial will help to answer a patient important question with significant clinical equipoise.

## Supporting information

S1 Checklist SPIRIT checklist. (DOCX)

S1 Protocol (DOCX)
