# Supplementary material for: Duration of face down positioning following full-thickness macular hole repair: A protocol for a randomized pilot study
Source: PLoS One. 2024 Aug 20;19(8):e0304566. doi: 10.1371/journal.pone.0304566 (PMC11335099; doi:10.1371/journal.pone.0304566)
Supplement: S1 Protocol — (DOCX) [file pone.0304566.s002.docx]

**Title:** Duration of Face Down Positioning Following Full-Thickness Macular Hole Repair: A Randomized Feasibility Study

**Supervisory Committee:**

**Corresponding Author and Primary Supervisor:** Dr. Varun Chaudhary, Hamilton Regional Eye Institute, St. Joseph’s Healthcare Hamilton King Campus, 2757 King Street East, Room 2500, Hamilton, ON, L8G 5E4. ORCID ID: 0000-0002-9988-4146.

**Co-supervisor:** Dr. Forough Farrokhayr, Department of Surgery, McMaster University ORCID ID: 0000-0001-9928-9016

**Funding:** None

**Introduction:**

*Background and rationale:*

Full-thickness macular holes (FTMH) are defects in the fovea involving all neural retinal layers.(1) They reduce patients’ visual acuity (VA) and have a significant impact on patients’ quality of life.(2) Left untreated, patients experience progressive loss of central visual acuity.(3) The current standard of repair for FTMHs is pars plana vitrectomy (PPV) with or without peeling of the internal limiting membrane (ILM), the use of intraocular gas tamponade and post-operative face down positioning (FDP).(1,3)

Face-down positioning (FDP) is the standard recovery posture for patients post PPV for FTMH. This however, can be physically challenging considering idiopathic FTMH occur most frequently in the elderly.(4) Previous investigations have demonstrated that over 50 percent of patients have described FDP as either difficult or very difficult.(5,6) Moreover, rare but serious systemic complications such as pulmonary embolism or ulnar nerve palsies have been reported after FDP.(7) Consequently, suggestions have been made to reduce the duration of FDP or eliminate FDP completely.(8,9). Presently, there is no consensus regarding the ideal positioning requirements following FTMH repair. A 2020 Preference and Trends Survey performed by the American Society of Retina Specialists and completed by 1273 retinal specialists from around the world demonstrated significant variability in current practices.(10) While the vast majority of surgeons recommend FDP, the duration of FDP varied considerably; the most frequently recommended duration was between 5-7 days of FDP (39.5% of responses) followed by 3-4 days (29.3% of responses).

There is, however, biologic plausibility to support minimal or no FDP following surgery. The rationale for FDP is to ensure that the FTMH can be isolated from intraocular fluid thereby permitting subfoveal fluid absorption and closure of the FTMH.(2,11) However, previous investigations have demonstrated that the first 24 hours is the most important time period for isolating the FTMH from the intraocular fluid.(2) A large gas bubble even without FDP would be sufficient to achieve this.(2) In-keeping with this, a recent meta-analysis of randomized controlled trials demonstrated no difference between FDP and non-FDP with respect to FTMH closure.(12) In this investigation, there was a visual benefit to FDP however the confidence intervals included values of trivial clinical significance. (12)

Previous investigations evaluating quality of life outcomes have demonstrated that patients prefer non-FDP; Veith et al. found that patients rated FDP as significantly more uncomfortable compared to non-FDP (p=0.010) and that patients found FDP to significantly impair sleep quality (p=0.001).(13) Tadayoni et al. found that when they asked patients to rate the painfulness of positioning on a visual analog scale (0-10), pain was rated 6.52 ± 2.48 and 2.53 ± 2.6 in a FDP and alleviated positioning group respectively.(7) Patient preferences are also reflected by the fact that compliance has been demonstrated to be lower in FDP groups compared to non-FDP,(7) and by the fact that compliance following PPV declines over time.(14) An investigation examining compliance during the first 3 days post-operatively found significantly decreased compliance each day after surgery. (14)

Given the lack of guidelines on the topic, the corresponding heterogeneity in practice patterns, the lack of biologic rationale, and the disabling nature of FDP for patients, there is a need to better assess key patient outcomes with different FDP durations. As such, this investigation by means of a prospective randomized controlled feasibility trial will compare 3-days of FDP to 7-days of FDP following PPV for FTMH. The population examined will be consecutive patients who present with an idiopathic FTMH to the Hamilton Regional Eye Institute. The intervention will be PPV with ILM peel followed by 3-days of FDP which will be compared to PPV with ILM peel followed by 7-days of FDP. The primary outcome will be the feasibility of completing a larger scale trial. Secondary outcomes will be focused on functional, structural, and quality of life outcomes. All outcomes will be examined at 3-months post-surgery.

*Objectives:*

Our primary objective will be focused on assessing feasibility. Feasibility will be assessed by measuring the recruitment rate, retention rate, and completion rate at 3-months following surgical repair as well as the time taken to recruit our desired sample size. Our secondary outcomes will include a) macular hole closure rate at 3-months following surgery; two independent readers, masked to treatment allocation, will independently grade the outcome in each instance as closed, or open. Outcomes classified as open will be further divided into open and flat (without a cuff of subretinal fluid), or open and elevated (with a cuff of subretinal fluid). b) best-corrected visual acuity at 3-months post-surgery, c) patient self-administered vision specific validated quality of life measure, the 25-item National Eye Institute Visual Function Questionnaire (NEI VFQ-25) and a validated quality of life measure, the Quality of Life Scale (QOLS), d) compliance with FDP as assessed utilizing self-administered questionnaires three times per day during the duration of FDP, and e) complication rates (endophthalmitis, retinal detachment, vitreous hemorrhage) as assessed by a masked physician. The interpretation of findings will be: a) the full-scale trial is feasible with no changes to the protocol; b) the full scale trial is feasible with small changes to the protocol; c) the full scale trial is infeasible. The study will be considered feasible if the retention and completion rates are > 80%. The study will be considered feasible with small changes if either the retention or completion rate is <80% and the study will be considered infeasible if the completion and retention rates are <80%.

*Trial design*

This study will be a single-centered, parallel group randomized controlled feasibility trial. Patients will be randomized 1:1 between groups.

**Methods:**

*Study Setting*

The study will be conducted at the Hamilton Regional Eye Institute located at St. Joseph’s Hospital in Stoney Creek Ontario. The study protocol is reported in line with the Standard Protocol Items: Recommendations for Interventional Trials (SPIRIT) statement.(15) Ethics approval for this project will be obtained through the Hamilton Integrated Research Ethics Board (HiREB). This study will adhere to the tenets of the Declaration of Helsinki.

*Eligibility criteria:*

All consecutive patients with an idiopathic FTMH with a symptom duration of less than 6 months who agree to participate will be included in this investigation. Patients with macular hole minimum diameter >1000 μm, a history of high myopia (> -6), traumatic macular hole, amblyopia, retinal vein occlusion, inflammatory eye diseases, or who were found to have a retinal tear during either the pre-operative assessment or intraoperatively will be excluded.

*Surgical technique:*

In both groups, patients will undergo standard three-port, 25-gauge PPV by one experienced retina specialist (Dr. VC). If epiretinal membranes are present, they will be peeled. The ILM will be stained utilizing indocyanine green (ICG) and will be subsequently peeled. Fluid-air exchange and a complete air-gas exchange will be performed utilizing sulfur hexafluoride (SF_6_ 18%) gas. The patients in both groups will be advised to posture face down immediately following surgery.

*Description of Interventions:*

Patients will be randomized 1:1 to the 3-day FDP and 7-day FDP groups. Patients will be advised to maintain the FDP for 50-minutes of each hour. They will be advised that during their 10-minute break each hour, they should avoid face-up positioning. FDP will be advised during both waking and sleeping hours.

*Data collection methods:*

After enrollment, patients will undergo standardized comprehensive ophthalmic examination including slit lamp examination, VA assessment, and measurement of intraocular pressure. VA will be measured with Snellen visual acuity. Standardized examinations along with OCT (either the Cirrus HD-OCT, Carl Zeiss Meditec, Dublin Ca or the Heidelberg Spectralis OCT, Franklin, MA depending on availability to the research assistant) will be performed within the 2-weeks prior to surgery and at the following post-operative times: 1-month, and 3-months. Patients will complete the NEI-VFQ-25 and the QOLS measures at baseline, and at 3-months following surgery. Compliance will be assessed utilizing a self-administered questionnaire in which patients will be asked to rate their compliance on a scale of 1-10 upon waking, at midday and before they go to sleep. Complication rates, including rates of retinal detachment, endophthalmitis and vitreous hemorrhage will be assessed by a physician at the 1-week, 1-month, and 3-month follow-up assessments.

*Data management:*

Data will be collected utilizing the secure online platform, Research Electronic Data Capture (REDCap). (16). Patients will be assigned a unique study number for which their information will be linked to. In addition to the information that will be collected as part of each ophthalmic examination, the following will be collected: the patient’s age, sex, duration of symptoms, past medical history, and past ocular history.

*Sample Size:*

The primary outcome of this investigation is to assess feasibility, as such, a power calculation to determine the sample size was not performed.(17–19) A literature review was undertaken to help inform the optimal sample size; no consensus was found with recommendations ranging from 24 to 50 described in the literature.(20–22) A previous RCT evaluating different positioning following PPV for FTMHs experienced a 10.2% attrition and thus we similarly estimate a 10% attrition rate in this investigation.(23) As such, we have decided to proceed with a sample size of 40 patients to inform our outcomes. We estimate based on previous rates of FTMH at our institution that there will be 4 new eligible patients each week. Previous studies at our institution have experienced a recruitment rate of 33%. Thus, we estimate that it will take approximately 30 weeks to achieve our target sample size.

Utilizing a sample size of 40, we will be able to estimate an 80% retention and completion rate to within a 95% confidence interval of +/-12.4% and a 33% recruitment rate to within a 95% confidence interval of +/- 14.6%.(24)

*Participant Timeline:*

Please note, this timeline may be modified depending on when we receive final ethics approval. Ethics approval will be submitted in February 2023. We estimate based on recruitment duration of previous studies at our centre that we will be able to recruit an average of 1.33 patients per week into this investigation. As such, we estimate that recruitment will take place from July 2023 until January 2024. We estimate that data collection will occur from July 2023 until April 2024.

Data analysis and manuscript preparation will occur in the summer of 2024 will the results of this investigation submitted to a peer-reviewed journal by the end of 2024.

*Recruitment:*

Participant recruitment will take place at the Hamilton Regional Eye Institute at the time of FTMH diagnosis. All patients will be informed regarding the risks, benefits, and alternatives to the surgical procedure and the different post-operative positioning durations. All patients will sign an informed consent form prior to enrollment into the study and before any measurement related to the study will be performed.

*Randomization and Masking*

Patients will be randomized 1:1 to the 3-day FDP or 7-day FDP groups utilizing permuted block randomization with blocks of 4, 6, and 8. Randomization will be performed prior to surgery; however, the surgeon will be made aware of the patient’s treatment allocation immediately following surgery. At this point the surgeon will inform the patient in the recovery room regarding their treatment allocation. Allocation concealment will be maintained utilizing REDCap. All staff performing VA assessments and obtaining OCT images will be masked to treatment allocation. OCT grading of FTMH closure will be performed by two masked readers and disagreements will be resolved by a third masked reader. The participants themselves and the clinical teams managing their care will be unmasked.

*Statistical methods and data analysis:*

Regarding the primary outcomes, the recruitment rate will be calculated as the number of patients randomized / the number of eligible patients screened. The retention rate will be defined as the number of patients who complete the follow-up measures at 3-months / the number of enrolled participants. Completion rates for data collection will be defined as the number of complete datasets for each of the outcome measures at 3-months / number of participants enrolled in the study. The recruitment time will be defined as the time taken in days to recruit 40 participants.

For the secondary outcomes, analyses will be performed on an intention-to-treat basis. If any outcome data are missing, we will assume data are missing at random and analyse data from available subjects. Continuous variables will be expressed as means (± standard deviation) and categorical variables will be expressed as percentages. Continuous variables will be compared utilizing the student’s t-test or Mann-Whitney U test based on normality. Categorial variables will be compared utilizing the Chi-Squared Test. Fisher’s Exact Tests were performed for expected values less than five. A p-value of <0.05 will be considered statistically significant. All estimations will be reported with 95% confidence intervals. All analyses will be performed using SPSS (version 23, Armonk, NY).

*Data monitoring:*

In-keeping with previous similar investigations,(23) no formal Data Monitoring Committee has been created given the relatively short time span of follow-up, and minimal clinical risks. However, an interim assessment will be performed once recruitment is 50% complete. If at this point the difference in FTMH closure rates between groups crosses the 95% confidence interval of the clinically significant noninferiority margin of 15% as determined by previous investigations assessing FTMH closure,(7,23) the study will be terminated.

*Declaration of Interests:*

KN: Dr. Nanji has no conflicts of interests.

VC: Dr. Chaudhary reports acting as an advisory board member, grants and other from Novartis, acting as an advisory board member, grants and other from Bayer, grants from Allergan, and acting as an advisory board member, and grants from Roche.

FF: Dr. Farrohkhyar has no conflicts of interest to disclose.

*Dissemination policy:*

The results of this study will be shared through peer-reviewed journals and conferences. All authors will adhere to the guidelines suggested by the International Committee of Medical Journal Editors Recommendations for the Conduct, Reporting, Editing, and Publication of Scholarly Work in Medical Journals. No professional writers will be used.

References:

1. Duker JS, Kaiser PK, Binder S, De Smet MD, Gaudric A, Reichel E, et al. The International Vitreomacular Traction Study Group classification of vitreomacular adhesion, traction, and macular hole. Ophthalmology [Internet]. 2013 Dec [cited 2022 Jan 18];120(12):2611–9. Available from: https://pubmed-ncbi-nlm-nih-gov.libaccess.lib.mcmaster.ca/24053995/

2. Forsaa VA, Lindtjørn B, Kvaløy JT, Frøystein T, Krohn J. Epidemiology and morphology of full-thickness macular holes. Acta Ophthalmol [Internet]. 2018 Jun 1 [cited 2022 Jan 18];96(4):397–404. Available from: https://onlinelibrary-wiley-com.libaccess.lib.mcmaster.ca/doi/full/10.1111/aos.13618

3. Flaxel CJ, Adelman RA, Bailey ST, Fawzi A, Lim JI, Vemulakonda GA, et al. Idiopathic Macular Hole Preferred Practice Pattern®. Ophthalmology [Internet]. 2020 Feb 1 [cited 2022 Feb 27];127(2):P184–222. Available from: http://www.aaojournal.org/article/S0161642019320937/fulltext

4. Cullen R. Macular hole surgery: helpful tips for preoperative planning and postoperative face-down positioning - PubMed. J Ophthalmic Nurs Technol [Internet]. 1998 Sep [cited 2022 Jan 18];17(5):179–81. Available from: https://pubmed-ncbi-nlm-nih-gov.libaccess.lib.mcmaster.ca/9919125/

5. Madgula IM, Costen M. Functional outcome and patient preferences following combined phaco-vitrectomy for macular hole without prone posturing. Eye 2008 228 [Internet]. 2007 Apr 13 [cited 2022 Jan 18];22(8):1050–3. Available from: https://www.nature.com/articles/6702835

6. Seno Y, Shimada Y, Mizuguchi T, Tanikawa A, Horiguchi M. COMPLIANCE WITH THE FACE-DOWN POSITIONING AFTER VITRECTOMY AND GAS TAMPONADE FOR RHEGMATOGENOUS RETINAL DETACHMENTS. Retina [Internet]. 2015 [cited 2022 Jan 18];35(7):1436–40. Available from: https://pubmed-ncbi-nlm-nih-gov.libaccess.lib.mcmaster.ca/25748281/

7. Tadayoni R, Vicaut E, Devin F, Creuzot-Garcher C, Berrod JP, Le Mer Y, et al. A randomized controlled trial of alleviated positioning after small macular hole surgery. Ophthalmology [Internet]. 2011 Jan [cited 2022 Jan 18];118(1):150–5. Available from: https://pubmed-ncbi-nlm-nih-gov.libaccess.lib.mcmaster.ca/21035869/

8. Masuyama K, Yamakiri K, Arimura N, Sonoda Y, Doi N, Sakamoto T. Posturing Time after Macular Hole Surgery Modified by Optical Coherence Tomography Images: A Pilot Study. Am J Ophthalmol. 2009 Mar 1;147(3):481-488.e2.

9. Dhawahir-Scala FE, Maino A, Saha K, Mokashi AA, McLauchlan R, Charles S. To posture or not to posture after macular hole surgery. Retina [Internet]. 2008 Jan [cited 2022 Jan 18];28(1):60–5. Available from: https://pubmed-ncbi-nlm-nih-gov.libaccess.lib.mcmaster.ca/18185139/

10. Preferences and Trends (PAT) Survey - The American Society of Retina Specialists [Internet]. [cited 2021 Jul 27]. Available from: https://www.asrs.org/asrs-community/pat-survey

11. Smiddy WE, Flynn HW. Pathogenesis of macular holes and therapeutic implications. Am J Ophthalmol [Internet]. 2004 [cited 2022 Jan 18];137(3):525–37. Available from: https://pubmed-ncbi-nlm-nih-gov.libaccess.lib.mcmaster.ca/15013877/

12. Chaudhary V, Sarohia GS, Phillips MR, Zeraatkar D, Xie JS, Nanji K, et al. Role of Positioning after Full-Thickness Macular Hole Surgery: A Systematic Review and Meta-Analysis. Ophthalmol Retin [Internet]. 2022 Jul [cited 2022 Dec 26]; Available from: https://pubmed-ncbi-nlm-nih-gov.libaccess.lib.mcmaster.ca/35781067/

13. Veith M, Vránová J, Němčanský J, Studnička J, Penčák M, Straňák Z, et al. Surgical Treatment of Idiopathic Macular Hole Using Different Types of Tamponades and Different Postoperative Positioning Regimens. J Ophthalmol [Internet]. 2020 [cited 2022 Dec 27];2020. Available from: /pmc/articles/PMC7847326/

14. Suzuki K, Shimada Y, Seno Y, Mizuguchi T, Tanikawa A, Horiguchi M. Adherence to the face-down positioning after vitrectomy and gas tamponade: A time series analysis. BMC Res Notes [Internet]. 2018 Feb 20 [cited 2022 Dec 26];11(1):1–5. Available from: https://bmcresnotes.biomedcentral.com/articles/10.1186/s13104-018-3257-1

15. Chan AW, Tetzlaff JM, Gøtzsche PC, Altman DG, Mann H, Berlin JA, et al. SPIRIT 2013 explanation and elaboration: guidance for protocols of clinical trials. BMJ [Internet]. 2013 Jan 9 [cited 2022 Feb 20];346. Available from: https://www.bmj.com/content/346/bmj.e7586

16. Harris PA, Taylor R, Thielke R, Payne J, Gonzalez N, Conde JG. Research electronic data capture (REDCap)--a metadata-driven methodology and workflow process for providing translational research informatics support. J Biomed Inform [Internet]. 2009 Apr [cited 2023 Feb 25];42(2):377–81. Available from: https://pubmed-ncbi-nlm-nih-gov.libaccess.lib.mcmaster.ca/18929686/

17. Thabane L, Ma J, Chu R, Cheng J, Ismaila A, Rios LP, et al. A tutorial on pilot studies: the what, why and how. BMC Med Res Methodol [Internet]. 2010 [cited 2021 Nov 23];10:1. Available from: /pmc/articles/PMC2824145/

18. Arain M, Campbell MJ, Cooper CL, Lancaster GA. What is a pilot or feasibility study? A review of current practice and editorial policy. BMC Med Res Methodol [Internet]. 2010 Jul 16 [cited 2021 Nov 23];10(1):1–7. Available from: https://bmcmedresmethodol.biomedcentral.com/articles/10.1186/1471-2288-10-67

19. Lewis M, Bromley K, Sutton CJ, McCray G, Myers HL, Lancaster GA. Determining sample size for progression criteria for pragmatic pilot RCTs: the hypothesis test strikes back! Pilot Feasibility Stud [Internet]. 2021 Dec 1 [cited 2021 Nov 23];7(1):1–14. Available from: https://pilotfeasibilitystudies.biomedcentral.com/articles/10.1186/s40814-021-00770-x

20. Julious SA. Sample size of 12 per group rule of thumb for a pilot study. Pharm Stat [Internet]. 2005 Oct 1 [cited 2021 Nov 23];4(4):287–91. Available from: https://onlinelibrary.wiley.com/doi/full/10.1002/pst.185

21. Lancaster GA, Dodd S, Williamson PR. Design and analysis of pilot studies: recommendations for good practice. J Eval Clin Pract [Internet]. 2004 May [cited 2022 Feb 21];10(2):307–12. Available from: https://pubmed-ncbi-nlm-nih-gov.libaccess.lib.mcmaster.ca/15189396/

22. Sim J, Lewis M. The size of a pilot study for a clinical trial should be calculated in relation to considerations of precision and efficiency. J Clin Epidemiol [Internet]. 2012 Mar [cited 2021 Nov 23];65(3):301–8. Available from: https://pubmed.ncbi.nlm.nih.gov/22169081/

23. Bainbridge JWB, Pasu S, Bell L, Zenasni Z, Lanz D, Simmonds IA, et al. Facedown Positioning Following Surgery for Large Full-Thickness Macular Hole: A Multicenter Randomized Clinical Trial. JAMA Ophthalmol [Internet]. 2020 Jul 1 [cited 2022 Apr 18];138(7):725. Available from: /pmc/articles/PMC7206530/

24. Sim J, Lewis M. The size of a pilot study for a clinical trial should be calculated in relation to considerations of precision and efficiency. J Clin Epidemiol [Internet]. 2012 Mar [cited 2022 Feb 21];65(3):301–8. Available from: https://pubmed-ncbi-nlm-nih-gov.libaccess.lib.mcmaster.ca/22169081/
